# Validity and reliability of the Niigata PPPD Questionnaire in a Western population

**DOI:** 10.1007/s00405-023-08038-1

**Published:** 2023-06-02

**Authors:** Rodrigo Castillejos-Carrasco-Muñoz, Ana Belén Peinado-Rubia, Miguel Ángel Lérida-Ortega, Alfonso Javier Ibáñez-Vera, María Cruz Tapia-Toca, Rafael Lomas-Vega

**Affiliations:** 1 Integral Balance Unit, Otorhinolaryngological Institute of Madrid, Madrid, Spain; 2 Department of Health Sciences, University of Jaen, Campus Las Lagunillas, S/N, Building B3, Office 212, 23071 Jaen, Spain; 3 Sanitary Management Area North of Jaen, San Agustin Hospital, Linares, Spain

**Keywords:** Dizziness, Vertigo, Unsteadiness, Validation studies, Reliability

## Abstract

**Purpose:**

To analyze the psychometric properties of the Niigata Questionnaire (NPQ) for use in a European population with persistent postural-perceptual dizziness (PPPD).

**Methods:**

Observational study included 140 patients with different vestibular conditions. Construct validity, internal consistency and concurrent validity were analyzed. Intra-class correlation coefficient (ICC), standard error of measurement (SEM) and minimal detectable change (MDC) were calculated. Receiver operating characteristic (ROC) curve was used to test diagnostic values.

**Results:**

Of the 140 patients, 47 had a diagnosis of PPPD. Factorial analysis showed a single-factor structure and concurrent validity analysis showed strong correlations with other instruments. Cronbach alpha coefficients of 0.938 for the total score, 0.869 for the standing and gait subscale, 0.803 for the subscale of movements and 0.852 for the visual stimulation subscale were obtained. The reproducibility was substantial except for the standing subscale, which could be considered moderate. For the standing, movement and visual stimulation subscales and for the total score, the SEM was 3.27, 2.41, 2.50 and 6.63, respectively, and the MDC was 6.40, 4.72, 4.91 and 12.99, respectively. The NPQ total score showed an area under the curve (AUC) of 0.661, a sensitivity of 72.34 and a specificity of 55.91 for discriminating between PPPD and other vestibular disorders.

**Conclusions:**

The NPQ is feasible for use in a Western population and presents a uni-factorial structure, high internal consistency and strong correlation with other instruments. The reliability can be considered substantial. The NPQ has low accuracy in discriminating between subjects with or without PPPD.

**Supplementary Information:**

The online version contains supplementary material available at 10.1007/s00405-023-08038-1.

## Introduction

Persistent postural-perceptual dizziness (PPPD), also known as functional dizziness, is a chronic vestibular disorder recognized by the International Classification of Vestibular Disorders [1] and is characterized by one or more symptoms of dizziness, unsteadiness or non-spinning vertigo present on most days for 3 months or more. This functional condition is one of the most frequent clinical entities; it is the first cause of chronic dizziness and is positioned as the first cause of consultation in adulthood between 19 and 64 years in specialized centers [2, 3]. Its prevalence peak appears between 30 and 50 years of age, and in terms of distribution by sex, this condition is more likely to affect women, with a female-to-male ratio of 3:1.

Different vestibular events, such as benign paroxysmal positional vertigo (BPPV), acute unilateral vestibulopathy/vestibular neuritis, Meniere's disease or central events, such as vestibular migraine or stroke, can act as precipitants of this condition, although their presence is not necessary or mandatory [4]. Other syndromes, diseases and functional conditions have been identified that can act as triggers [5], as well as a certain predisposition in the healthy population to the development of this health problem [6].

PPPD patients usually have dizziness that during the early stages tends to fluctuate with periods of exacerbation and even remission. Subsequently, dizziness, although it continues to fluctuate in terms of the intensity with which the subject perceives it, will remain persistent [4]. The symptoms can be exacerbated by different demanding sensory conditions, such as mobile visual environments (busy streets, road traffic, etc.), highly structured visual environments or when visual references are far apart [5]. In the same way, the patient's active own movement of both the head and the body as a whole, as well as passive movement (means of transport, escalators, etc.) and upright movement, may cause an increase in the patient's symptoms [4].

Within therapeutic management, multimodal approaches have shown efficacy in the medium term, such as the combination of serotonergic medication, cognitive–behavioural psychotherapy and physiotherapy [7, 8]. With regard to the approach through physiotherapy, the protocols are based on desensitization models through neurosensory stimulation of increasing intensity [9, 10]. Due to the lack of well-defined procedures and doses, the protocols are dependent on each work team [9, 10]. Thus, the identification of these exacerbating factors plays an important role in defining what type of action through physiotherapy is most adapted.

With this scenario, a health measurement instrument has recently been developed for this problem. The Niigata PPPD Questionnaire (NPQ) is the first instrument developed to measure the severity of PPPD. The NPQ includes 12 items that yield a global score. Furthermore, the items are organized into three four-item subscales that specifically assess the three exacerbating factors [11]. The original validation study included the measurement of two psychometric properties, internal consistency and diagnostic validity, through ROC curves and the establishment of cut-off points to predict the presence of PPPD. However, other characteristics, such as factorial validity, concordant validity with other similar measures or reproducibility, have not been studied, nor has the value of the standard error of measurement (SEM) or the minimum detectable change (MDC) been obtained.

The NPQ is the first questionnaire developed specifically for the measurement of health in patients with PPPD that is increasingly used in the country in which it was developed. However, its psychometric characteristics have been insufficiently studied. Although the authors of the original article present an English version of the questionnaire, it has not been evaluated outside the borders of Japan. For this reason, this study considered the analysis of the main psychometric properties of the NPQ in a Western population. This work required the cross-cultural adaptation of the questionnaire from the Japanese language to the Spanish language for its use for the first time in a European population.

Our work aims to validate the NPQ for use in Western populations. We also aim to analyze the psychometric properties of the NPQ in a population of patients with vestibular disorders. Specifically, this study aimed to measure the construct validity, internal consistency, reproducibility and concurrent validity of the NPQ as well as its ability to discriminate between patients with PPPD and patients with other conditions.

## Materials and methods

### Design

An observational questionnaire validation study was performed according to the ethical Declaration of Helsinki and approved 03/15/2022 by the Ethics Research Committee of the University of Jaen (Spain) (reference code ENE.22/7.PRY).

### Participants

For the sample size calculation, it was considered the principle of using at least 10 subjects per item, with a minimum of 80 for validity studies [12]. Therefore, considering that the NPQ is composed of 12 items, a total sample of almost 120 participants was needed. Regarding the test–retest analysis, a total of 30 participants were considered [12]. Potential participants were recruited from a cohort of consecutive subjects who attended the Otorhinolaryngological Institute of Madrid between March and November of 2022. The participants were recruited based on their diagnosis.

The selection criteria included participants over 18 years old diagnosed with vestibular pathology. The exclusion criteria were psychiatric or neurological problems that could interfere in the comprehension or accomplishment of the required forms. An otorhinolaryngologist with more than 15 years of experience performed the participant selection and diagnosis.

The enrolled participants were personally informed by the otorhinolaryngologist about the study, their right to withdraw at any time without need to give explanations and the possibility of asking about any concern. Informed consent was provided by all participants before they were given the assessment notebook containing all the questionnaires and required evaluations. The same member of the research team was present during the assessment to clarify any doubt concerning the questionnaires. For the retest, the participants received a link on their mobile phone through email or message where they found the NPQ exported to a Google Form^®^ that they could easily fill in. The data from the online questionnaire were anonymously coded through a Microsoft Excel^®^ sheet and added to the rest of the data. Only the first 30 participants to complete the retest were considered.

### Cross-cultural adaptation and translation

Following the recommendations of the IQOLA Project for translating health status questionnaires and evaluating their quality [13], the original Japanese NPQ was independently translated into Spanish by two bilingual native speakers with the help of two clinical experts in the field. Translators and researchers had to reach a consensus about the final version of the forward translation. The next step was to translate the Spanish consensus version backward to Japanese, which was performed by two bilingual experts. This last re-translated version was compared by the researchers with the original Japanese version to verify the conceptual, linguistic, semantic and technical equivalence. The last step was to test the Spanish version of the questionnaire viability using it on 20 subjects to assess that they could understand the instructions, items and answers. All the subjects were supervised while completing the Spanish NPQ to detect any trouble. The average time required for completing the NPQ was of 3–5 min.

### Variables

The following sociodemographic data were recorded: age (years), gender (male or female), weight (kilograms) and height (meters) (with a Detecto Model 2391 scale with a height rod), body mass index, marital status, income level, educational level and medical diagnosis. Subjects were also required to indicate if they had suffered any fall in the last 12 months and the number of falling episodes in that period. A different researcher from the one performing the diagnosis and participant selection was in charge of collecting and coding this information.

The Activities-Specific Balance Confidence Scale was used in its standard (ABC-16) [14] and short version (ABC-6) [15] to assess the risk of falling in vestibular patients. This scale is composed of 16 items that ask about the degree of confidence in balance while performing some specific daily tasks. The items are scored a scale from 0% (no confidence) to 100% (full confidence), so the total scores are also between 0 and 100%, resulting in the average of the items. Values under 67% are considered sensitive and specific in predicting falls and reducing the independence of subjects in a considerable way [16]. The short version of the scale (ABC-6) was extracted from items 5, 6 and 13–16 of the main version, showing good internal consistency, moderate test–retest reliability and a minimum detectable change of 20 points. Its concurrent validity with other tests that evaluate dizziness disability is strong [15], so even more researchers and clinicians are tending to use this short version.

To assess the impact of dizziness and balance in participants, the Dizziness Handicap Inventory (DHI) was chosen [17]. This tool includes 25 items that are answered “yes”, “no” or “sometimes”, scoring 4, 0 or 2, respectively. The DHI includes three dimensions: functional (nine items), physical (seven items) and emotional (nine items). The scores of the different dimensions are categorized as “no disability” if the scores on the functional and emotional dimensions are under 15 and the scores on the physical dimension are under 10, “moderate disability” if the scores on the functional and emotional dimensions range from 15 to 24 and the scores of the physical dimension range from 10 to 16, and “severe disability” if the scores on the functional and emotional dimensions range from 24 to 36 and scores on the physical dimension range from 17 to 28. A global total score is also considered, where “mild disability” is indicated by a score from 0 to 30, “moderate disability” is indicated by a score from 31 to 60 and “severe disability” is indicated by a score from 61 to 100 [17].

The Visual Vertigo Analog Scale (VVAS) was also used, which asks the subject about nine challenging conditions that could induce dizziness [18]. Each condition is scored by marking a line on a 10-cm segment whose endpoints are marked as “0 = no dizziness” and “10 = maximum dizziness”. According to the original version, this scale presents good internal consistency, moderate–strong concurrent validity with the DHI and the ability to discriminate among those subjects with vestibular disorders and those with a healthy vestibular condition [19].

The Central Sensitization Inventory (CSI) was used to assess the presence of central sensitization in the participants. This questionnaire includes a wide range of emotional and somatic symptoms commonly related to patients with central sensitization syndromes [20]. The score ranges from 0 to 100, where a greater value means a greater intensity of symptoms. The cut-off point for determining the presence of central sensitization was established as 40 points [20].

General health condition was measured with the SF-12, a self-administered questionnaire that combines 12 items about mental and physical health. It is the short version of the SF-36, with the total score ranging from 0 (poor quality of life) to 100 (best quality of life) (Vilagut et al. [21]).

### Data analysis

Data were described by means and standard deviations for continuous variables and by frequencies and percentages for categorical variables. The normality of the samples was analyzed with the Kolmogorov‒Smirnov test, and homoscedasticity was analyzed with the Levene test.

The construct validity was analyzed by means of factorial analysis. Factor extraction was performed using principal component analysis, and varimax-type rotation with Kaiser was planned. The relevance and feasibility of the analysis was measured with the Kaiser‒Meyer‒Olkin (KMO) test, with the Bartlett sphericity test and with the calculation of the determinant of the correlation matrix.

Internal consistency was analyzed with Cronbach's α coefficient, which is used to evaluate the extent to which the items of an instrument are correlated with each other [22]. A Cronbach alpha coefficient less than 0.70 indicates poor reliability, a coefficient between 0.70 and 0.90 indicates good reliability and a coefficient greater than 0.90 indicates redundancy [23].

Test–retest reliability was analyzed using the Shrouth and Fleiss intra-class correlation coefficient (ICC) using an absolute agreement, two-way random-effects model [24]. Reliability was categorized as poor when the ICC was < 0.40, moderate when the ICC was between 0.40 and 0.75, substantial when the ICC was between 0.75 and 0.90, and excellent when the ICC was > 0.90[25]. The standard error of measurement (SEM) was calculated by the formula ${\text{SEM}} = \sigma_{{{\text{base}}}} \times \sqrt {\left( {1 - {\text{CCI}}} \right)}$, and the minimum detectable change (MDC) was obtained as the 95% confidence interval of the SEM (MDC95) with the formula ${\text{MDC}}_{95} = Z \times \sigma_{{{\text{base}}}} \times \sqrt {\left( {\text{1 - ICC}} \right)}$, where “σbase” is the standard deviation of the pretest measures, “ICC” is obtained from the test–retest reliability and “*Z*” is the numerical value of the 95% confidence interval (CMD95), which is 1.96.

Pearson’s correlation coefficient (*r*) was used to test concurrent validity. Following Cohen’s criteria [26], the correlation is considered "strong" when r is greater than 0.50; if r is between 0.50 and 0.30, the correlation is considered “moderate”; and if r is less than 0.30, the correlation is considered “insignificant”.

To analyze the diagnostic validity of NPQ, ROC (receiver operating characteristic) curve analysis was performed [27]. The area under the ROC curve (AUC) was used to analyze the capacity of NPQ to discriminate between subjects with PPPD and other vestibular conditions. A test is considered capable of discriminating between two groups of subjects when the AUC value is > 0.50; an AUC value between 0.50 and 0.70 indicates that the test has a “low accuracy”; when the value is between 0.70 and 0.90 the test has a “good accuracy”; and when the value exceeds 0.90, the test has a “high accuracy” [28].

Through the analysis of ROC curves, the optimal cut-off points of the NPQ score that determine the threshold of functional alteration were obtained, from which the predictive values could be calculated. Sensitivity was defined as the proportion of PPPD subjects who tested positive for NPQ scores. Following this reasoning, specificity was calculated as the proportion of subjects without PPPD diagnosis who were negative on the NPQ score. Positive predictive value (PPV) was defined as the proportion of subjects with a positive NPQ score who had PPPD. Negative predictive value (NPV) was defined as the proportion of patients with a negative NPQ score who had diagnoses other than PPPD.

For data analysis, IBM SPSS Statistics for Windows, version 26 (IBM Corp., Armonk, N.Y., USA) and MedCalc^®^ Statistical Software version 20.211 (MedCalc Software Ltd, Ostend, Belgium; https://www.medcalc.org; 2023) were used. We used a confidence level of 95% (alpha error of 5%).

## Results

**Fig. 1:**
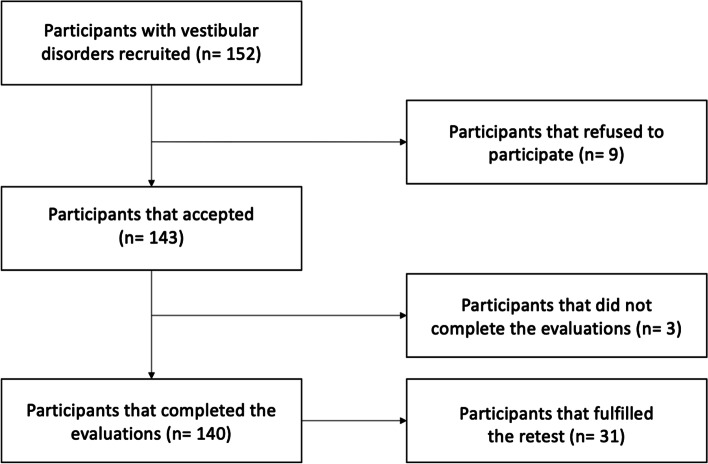
Flow diagram of participants

The study involved 140 subjects (Fig. 1), among whom 47 had a diagnosis of PPPD following the stabilized criteria. In the group of “other” vestibular conditions, the most frequent diagnosis was benign paroxysmal positional vertigo (BPPV). The main characteristics of the final sample are shown in Table 1. Additionally, in 31 patients, a second evaluation could be obtained approximately between one and two weeks after the first evaluation.

**Table 1:** Sociodemographic characteristics of the sample

Variable	Category	Mean	SD	Frequency	Percentage
Age		49.46	13.58		
Height		168.76	9.48		
Weight		70.45	13.85		
Body mass index		24.60	3.53		
Sex	Female			78	55.7
	Male			62	44.3
Study level	Primary			8	5.8
	Secondary			29	20.9
	University			59	42.4
	University completed			43	30.9
Economic level	< 20.000 Euros/year			29	21.0
	> 20.000 Euros/year			109	79.0
Civil status	Single			40	29.0
	Married			80	58.0
	Divorced			10	7.2
	Widowed			8	5.8
Diagnostic	PPPD			47	33.6
	BPPV			52	37.1
	Bilateral vestibulopathy			6	4.3
	Meniere’s disease			14	10.0
	Vestibular migraine			2	1.4
	Acute vestibular syndrome			3	2.1
	Superior semicircular canal dehiscence			1	0.7
	Unilateral vestibulopathy			4	2.9
	Head trauma			1	0.7
	Migraine			7	5.0
	Vestibular schwannoma			2	1.4
	Vestibular dysplasia			1	0.7

**Fig. 2:**
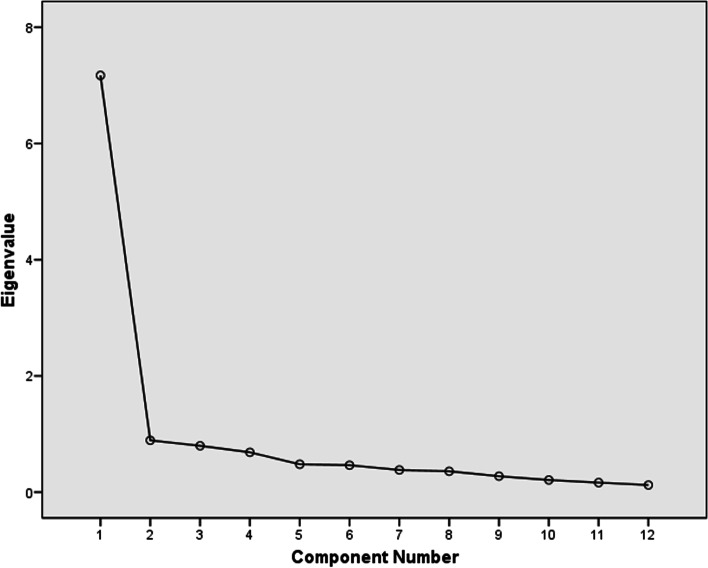
Scree Plot

The factorial validity measured by principal components analysis showed a single factor structure (Fig. 2) that explained approximately 60% of the total variance. The KMO = 0.896 was satisfactory, and the Bartlett sphericity test was significant (*X*_2_ = 1207,691, *p* < 0.001). The determinant of the matrix was very close to “zero”, which indicates that the correlation matrix between the NPQ items is very different from the identity matrix (absence of correlations between the items). All of these measurements indicated that the sample could be considered suitable for factor analysis.

The internal consistency analysis of the questionnaire taken globally yielded a Cronbach alpha coefficient = 0.938, which could indicate some redundancy of the items. Their analysis (Table 2) indicated that the elimination of any of them would slightly decrease the alpha value but without drastic changes. In the analysis of the subscales, the values can be considered good, with Cronbach’s alpha = 0.869 for the standing and gait subscale, Cronbach's alpha = 0.803 for the subscale of movements and Cronbach's alpha = 0.852 for the visual stimulation subscale. In practically all the cases, the scores that can be considered as good and not indicative of redundancy, the elimination of the items harmed the internal consistency of the subscale.

**Table 2:** Item analysis

	Average of the scale if the element is removed	Scale variance if element is removed	Corrected item-total correlation	Squared multiple correlation	Cronbach’s alpha if the element is removed
NPQ_Item1	19.50	250.007	0.596	0.511	0.937
NPQ_Item2	20.29	240.338	0.762	0.639	0.931
NPQ_Item3	20.67	242.639	0.753	0.703	0.931
NPQ_Item4	20.36	244.305	0.658	0.630	0.935
NPQ_Item5	20.55	241.314	0.688	0.584	0.934
NPQ_Item6	20.97	247.654	0.684	0.668	0.933
NPQ_Item7	20.59	242.575	0.695	0.709	0.933
NPQ_Item8	20.41	240.977	0.790	0.732	0.930
NPQ_Item9	20.53	243.129	0.787	0.690	0.930
NPQ_Item10	20.49	245.273	0.693	0.521	0.933
NPQ_Item11	20.46	235.071	0.816	0.799	0.928
NPQ_Item12	20.95	245.285	0.738	0.602	0.932

The reproducibility of the global score and subscales could be considered substantial except for the standing subscale, which could be considered moderate. Generally, the items showed moderate reproducibility except for items 4, 8 and 11, which could be considered substantial (Table 3). Taking into account the ICC found, as well as the pretest standard deviation, the SEM was 3.27, 2.41, 2.50 and 6.63, and the MDC was 6.40, 4.72, 4.91 and 12.99 for the standing, movement and visual stimuli subscales and the total score, respectively.

**Table 3:** Test re-test reliability measured by ICC values of the items, subscales and total score of the NPQ

ITEM	ICC	Lower	Upper	*p* value	Reproducibility
Item 1	0.623	0.225	0.818	0.004	Moderate
Item2	0.634	0.247	0.823	0.003	Moderate
Item 3	0.669	0.319	0.840	0.002	Moderate
Item 4	0.825	0.640	0.915	0.000	Substantial
Item 5	0.677	0.336	0.844	0.001	Moderate
Item 6	0.499	− 0.031	0.757	0.030	Moderate
Item 7	0.737	0.459	0.873	0.000	Moderate
Item 8	0.837	0.664	0.921	0.000	Substantial
Item 9	0.722	0.429	0.866	0.000	Moderate
Item 10	0.576	0.129	0.795	0.010	Moderate
Item 11	0.876	0.746	0.940	0.000	Substantial
Item 12	0.620	0.219	0.816	0.005	Moderate
Standing/walking	0.739	0.464	0.874	0.000	Moderate
Movement	0.829	0.649	0.917	0.000	Substantial
Visual stimuli	0.837	0.666	0.921	0.000	Substantial
Total score	0.852	0.696	0.929	0.000	Substantial

The concurrent validity analysis between NPQ scores and other variables of interest is shown in Table 4. The total score and subscale scores showed insignificant correlations with falls in the last 12 months and with the Physical Component Summary (PCS) of the SF-12. The correlation was moderate with the Mental Component Summary (MCS) of the SF-12, and there was a moderate correlation between the visual stimulation subscale and the measured central sensitization. The rest of the correlations were strong, showing good concurrent validity of the NPQ measures and DHI subscales, ABC scales, CSI and VVAS.

**Table 4:** Concurrent validity of the NPQ total score and subscales with balance and dizziness measures

	NPQ total score			Upright Posture/Walking			Movement			Visual Stimulation		
	*r* Pearson	*P* value	Validity	*r* Pearson	*p* value	Validity	*r* Pearson	*p* value	Validity	*r* Pearson	*p* value	Validity
ABC-6	– 0.691	< 0.001	Strong	– 0.674	< 0.001	Strong	– 0.662	< 0.001	Strong	– 0.601	< 0.001	Strong
ABC TOTAL	– 0.739	< 0.001	Strong	– 0.729	< 0.001	Strong	– 0.706	< 0.001	Strong	– 0.636	< 0.001	Strong
VVAS TOTAL	0.636	< 0.001	Strong	0.515	< 0.001	Strong	0.655	< 0.001	Strong	0.618	< 0.001	Strong
DHI Emotional	0.654	< 0.001	Strong	0.634	< 0.001	Strong	0.620	< 0.001	Strong	0.577	< 0.001	Strong
DHI Functional	0.735	< 0.001	Strong	0.698	< 0.001	Strong	0.705	< 0.001	Strong	0.659	< 0.001	Strong
DHI Physical	0.600	< 0.001	Strong	0.555	< 0.001	Strong	0.604	< 0.001	Strong	0.526	< 0.001	Strong
DHI TOTAL	0.751	< 0.001	Strong	0.714	< 0.001	Strong	0.726	< 0.001	Strong	0.666	< 0.001	Strong
CSI TOTAL	0.521	< 0.001	Strong	0.506	< 0.001	Strong	0.528	< 0.001	Strong	0.428	< 0.001	Moderate
PCS SF12	– 0.148	0.082	Insignificant	– 0.177	0.036	Insignificant	– 0.173	0.041	Insignificant	– 0.064	0.449	Insignificant
MCS SF12	– 0.386	< 0.001	Moderate	– 0.360	< 0.001	Moderate	– 0.372	< 0.001	Moderate	– 0.351	< 0.001	Moderate
Falls 12 Months	0.178	0.036	Insignificant	0.219	0.009	Insignificant	0.186	0.028	Insignificant	0.093	0.276	Insignificant

**Fig. 3:**
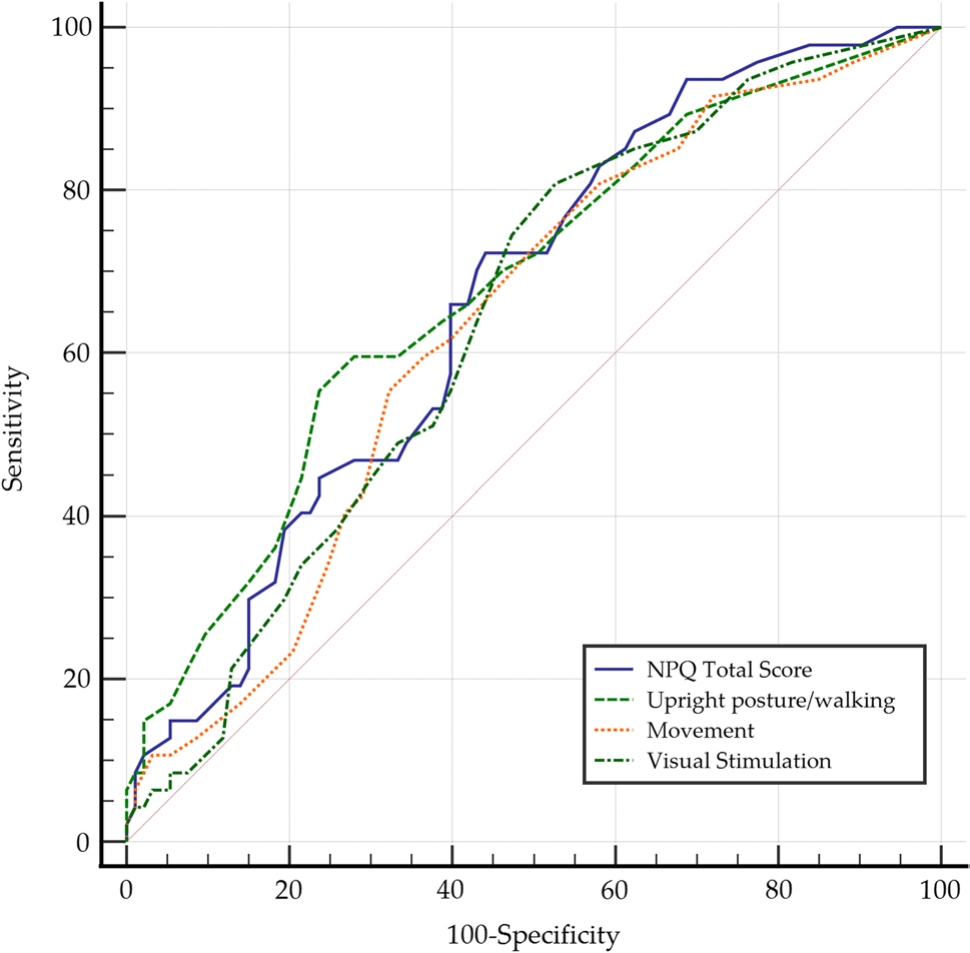
ROC curve analysis

The diagnostic validity of the NPQ total score and subscales is shown in Table 5, and the ROC curves are shown in Fig. 3. In general, the diagnostic capacity was statistically significant but could be classified as low accuracy or in the range of low to good accuracy for the total and subscale scores. Sensitivity was high for the total score and visual stimulation subscale, and the specificity was better for the standing and movement subscales.

**Table 5:** ROC curve analysis and values predicting PPPD from NPQ scores

Variable	Criterion	Sensitivity	95% CI	Specificity	95% CI	AUC	SE	95% CI
NPQ Total score	> 16	72.34	57.4– 84.4	55.91	45.2–66.2	0.661	0.047	0.577–0.739
Upright posture/walking	> 9	55.32	40.1–69.8	76.34	66.4–84.5	0.680	0.048	0.596–0.756
Movement	> 8	55.32	40.1–69.8	67.74	57.3–77.1	0.633	0.048	0.547–0.713
Visual stimulation	> 4	80.85	66.7–90.9	47.31	36.9–57.9	0.640	0.048	0.554–0.719

## Discussion

This study aimed to analyze the psychometric properties of the Niigata PPPD questionnaire for use in Western vestibular patients. The study was necessary for two fundamental reasons. First, there is an absence of specific instruments to determine the impact of the disease in patients with PPPD. Second, the psychometric properties of this instrument have never been studied, even in the original culture in which the questionnaire was validated. Our study provides NPQ data that have never been analyzed, such as concurrent validity with other measures of interest, and reproducibility with error of measurement and minimum detectable change.

In our study, the factorial analysis reported a unifactorial structure for the NPQ. These results are contradictory to those obtained by Chichiro Yagi et al. [29], which reported a structure in three factors that could be defined as a visual factor, Active-Motion Factor and Passive Motion/Standing Factor. The differences in the results of the two studies are surely due to the composition of the sample, since our factorial analysis was carried out in a population of different types of vertigo while Chichiro Yagi et al. had only patients with PPPDs. Future studies should investigate the structure of the NPQ in an adequate population of western subjects with PPPD.

Regarding the internal consistency analysis, our data are consistent with the original validation study that showed a Cronbach's alpha of 0.91 [11], similar to the value of 0.938 found by us. In both cases, it can be interpreted as the presence of a certain redundancy of the items taken globally [23], but whose effect is not observed in the subscales that present Cronbach's alpha values below 0.90 in both the Japanese version and ours.

With regard to diagnostic validity, our results are slightly worse than those of the original study [11]. Our findings suggest that the NPQ is very good at measuring the severity of the symptoms; however, its results as a PPPD diagnostic tool are poorer, which is secondary since it is not its main purpose. The reason for this observation could be the punctuation and the composition of the “control” group to compare, since our control group has more cases of migraine and vestibular migraine that have usually presented a high NPQ score and its subscales in a similar way than PPPD subjects. Nonetheless, Kitazawa et al. noted that the NPQ is the most useful tool for the diagnosis of PPPD when compared with the video head impulse test, posturography, rotatory chair test, the DHI, bithermal caloric test and cervical and ocular vestibular-evoked myogenic potential assessment [30].

Concerning concurrent validity, the NPQ showed a strong correlation with the ABC-16, ABC-6, VVAS and DHI, which are the main instruments used in the assessment of vestibular disorders. At this point and considering the high correlation between the NPQ and the CSI as well as the composition of our sample, we hypothesized that PPPD could follow a similar model to other central sensitization disorders. In this way, PPPD may be considered and treated as a functional disorder.

To our knowledge, since the publication of the scale in 2019, several studies have used the NPQ in the assessment of PPPD in Japanese patients. In addition to the study of Kitazawa [30] previously mentioned, Yamaguchi et al. used the NPQ to assess the effects of a treatment based on virtual reality in participants with PPPD, determining that participants improved their symptoms [31]. Similarly, Miwa and Kanemaru observed 15.5 points of improvement in the NPQ using traditional medicine in PPPD patients [32], which is greater than the MDC obtained by our study. Thus, these authors and future authors would benefit from our study, as we calculated the MDC for the NPQ, allowing a better and more precise interpretation of the results of every study that uses the NPQ. The same could be applied to the study of Eldøen et al., who assessed a website with vestibular rehabilitation videos for PPPD patients [33], and the study of Yagi et al., who considered gaze instability in PPPD [34]. Other studies used the NPQ to assess the relation between the presence of isolated otolith dysfunction and PPPD [35] or the presence of exacerbating factors for PPPD patients [36].

Regarding the use of the scale in clinical or research settings, our data show that the NPQ can be used both based on its overall score and the value of each subscale, which provides more specific information on the impact of the disease in different capacities of each patient. Although this impression is not justified by the factorial analysis that reports a one-dimensional item structure, it can be supported by the fact that the internal consistency analysis offers optimum values for each subscale. Additionally, the strong correlation with other scales that measure dysfunction due to dizziness or confidence in balance should give us the necessary confidence to use the NPQ as a specific instrument when working with subjects with PPPD.

Another key aspect of our study is obtaining the SEM and MCD values that report the magnitude of the change that clinicians or researchers can interpret as a simple measurement error or as a substantial change in the patient's state of health. Finally, our analysis of ROC Curves does not justify the use of the NPQ as a screening instrument, since poor predictive values have been obtained, so we recommend the diagnosis of PPPD based on the criteria established up to now.

This study has several limitations. First, the validity of our results can be applied to the European population that speaks Spanish without being properly extrapolated to other Western languages. Although the data have been extracted from a sufficiently diverse population of vestibular patients and with a valid total sample to obtain statistical results, the total number of subjects has been low for some categories, which prevents drawing conclusions for specific pathologies beyond PPPD. Finally, although our study analyses psychometric properties that had not been evaluated in the original version, some additional properties remain unanalysed, such as sensitivity to change or validity to discriminate between different vestibular pathologies.

In view of the results of our work, it can be concluded that the NPQ is a feasible instrument for use in the Western culture population. The NPQ presents a unifactorial structure with a high internal consistency that could indicate a certain redundancy of the items. However, the three subscales that make up the NPQ present good internal consistency but without redundancies between items. The test–retest reliability of the overall score and of the subscales can be considered substantial except for the standing subscale, which can be considered moderate. This reliability provides a measurement error of 6 points and a minimum detectable change of 13 points for the overall score. Both the global score and the NPQ subscales have strong concurrent validity with the magnitude of dizziness measured with the DHI and its subscales, balance confidence measured with the ABC-16 and ABC-6, with the central sensitization measure CSI, and with the vertigo visual analog scale. However, the correlation was moderate with the MCS of the SF-12 and negligible with falls in the last 12 months and with the PCS of the SF-12. The overall score and the NPQ subscales have low accuracy in discriminating between subjects with or without PPPD, possibly because the comparison group was made up of very diverse pathologies that correspond to high scores on the NPQ. Future research should analyze the values of the NPQ and its subscales in different subgroups of patients with vestibular and neurological pathologies. The sensitivity of this instrument to detect significant changes in the health of patients should also be analyzed.

### Supplementary Information

Below is the link to the electronic supplementary material.

Supplementary file1 (DOCX 15 KB)

## Data Availability

The data of this study is available under reasonable request to the corresponding author.
