# Supplementary material for: Validity and reliability of the Niigata PPPD Questionnaire in a Western population
Source: Eur Arch Otorhinolaryngol. 2023 Jun 2;280(12):5267–76. doi: 10.1007/s00405-023-08038-1 (PMC10620260; doi:10.1007/s00405-023-08038-1)
Supplement: Supplementary file 1 — Supplementary file1 (DOCX 15 KB) [file 405_2023_8038_MOESM1_ESM.docx]

**Cuestionario de Mareo Postural Perceptivo Persistente Niigata**

Este cuestionario está diseñado para identificar las dificultades en las actividades de la vida diaria debidas a sus síntomas de mareo. Por favor, rodee con un círculo el número que mejor describa cómo estas actividades se han visto afectadas durante la última semana. Siendo el “0” que puede realizar la actividad sin ninguna dificultad y el “6” que evita realizar la actividad por ser insoportable. Si sus síntomas fluctúan, califique y marque con un círculo los síntomas de mayor intensidad de la última semana.

P1. Realizar movimientos bruscos, tales como ponerse de pie o girar la cabeza repentinamente: 0 1 2 3 4 5 6

P2. Mirar las estanterías de los supermercados y grandes superficies:

0 1 2 3 4 5 6

P3. Caminar a su propio ritmo, a su velocidad normal: 0 1 2 3 4 5 6

P4. Ver imágenes en la televisión o en las películas en las que haya mucho movimiento:

0 1 2 3 4 5 6

P5. Ir en coche, autobús o tren: 0 1 2 3 4 5 6

P6. Permanecer sentado en una silla sin respaldo ni reposabrazos: 0 1 2 3 4 5 6

P7. Permanecer de pie sin ningún apoyo ni contacto: 0 1 2 3 4 5 6

P8. Mirar una pantalla de ordenador o teléfono en la que hay desplazamiento de las imágenes: 0 1 2 3 4 5 6

P9. Realizar actividades como las tareas domésticas o hacer ejercicio ligero: 0 1 2 3 4 5 6

P10. Leer la letra pequeña en los libros y/o los periódicos: 0 1 2 3 4 5 6

P11. Caminar a un ritmo rápido: 0 1 2 3 4 5 6

P12. Subir en un ascensor o en una escalera mecánica: 0 1 2 3 4 5 6

Eso esto todo. Muchas gracias.

Por favor, NO rellene ningún campo aquí

Puntuación Sensibilidad a postura erguida y marcha (=3+6+7+11) /24

Puntuación Sensibilidad al movimiento activo y pasivo (=1+5+9+12) puntos /24
Puntuación Sensibilidad a entornos Visuales móviles o muy estructurados (=2+4+8+10) puntos /24

Puntuación global /72
